# Enucleated globes with choroidal melanoma: A retrospective histopathological study and correlation with cytogenetic profile in 2 eye centers

**DOI:** 10.1016/j.amsu.2020.05.003

**Published:** 2020-06-01

**Authors:** Hind M. Alkatan, Abdullah Aoun Al Qahtani, Azza MY. Maktabi

**Affiliations:** a Ophthalmology Department, King Saud University Riyadh, Saudi Arabia; b Pathology Department, King Saud University, Riyadh, Saudi Arabia; c Surgical Vitreo-retina, Ophthalmology Department, Imam Abdulrahman Bin Faisal University, Dammam, Saudi Arabia; d Pathology & Laboratory Medicine Department, King Khaled Eye Specialist Hospital, Riyadh, Saudi Arabia

**Keywords:** Enucleation, Cytogenetic, Chromosome, Choroidal melanoma, Histopathology

## Abstract

**Background:**

Uveal melanoma is the commonest intraocular malignant tumor in adults and the choroid is the commonest involved location. It is more prevalent in Caucasians; however, the demographics are widely variable based on ethnicity. Histopathological features have been correlated to the cytogenetic profile, which we intend to report through the study of enucleated eyes with choroidal melanoma (CM).

**Materials and Methods:**

A retrospective review of 28 enucleated globes with CM in 2 tertiary eye centers (January 2000-December 2017). The tumors were histopathologically classified based on the 8th edition of the American Joint Committee on Cancer (AJCC). The histopathological risk factors and the AJCC classifications were correlated with Fluorescence in situ hybridization (FISH) for chromosomes 3 and 8 available results in 18/28 eyes.

**Results:**

We have included 28 patients with a mean age of 56 years, 13 males (46.4%) and 15 females (53.6%). None had lymph node involvement or metastatic disease. The tumor size was categorized as 3 and 4 in 68% of eyes. Half tumors were of spindle cell type and were associated with absent cytogenetic abnormality in chromosomes 3 and 8 (P=0.005). Closed vascular loops presence was significantly associated with abnormal chromosomes 3 and 8 (P=0.027).

**Conclusion:**

Patients in our area presented late with larger tumor size. The spindle cell CM was the commonest and correlated with negative FISH results, while the presence of closed vascular loops was a risk factor for abnormal FISH results hence expected worse prognosis. AJCC classification did not correlate well with our FISH results.

## Highlights

• Spindle cell type was the commonest in 28 enucleated globes with choroidal melanoma.
• Mean age was 61 years with slight female predominance in 54%.
• Large tumors (AJCC category sizes 3 and 4) were seen in 68% due to late presentation.
• Absent chromosomes 3 and/or 8 abnormality was significantly associated with spindle cell type (*P=0.005*).
• Chromosomal abnormality (3 and/or 8) was significantly associated with closed vascular loops (*P=0.027*).

## 1 Introduction

Uveal melanoma (UM) is the commonest primary intraocular malignancy in the adult population, affecting 4 to 7 per million in the United States per year [1]. It occurs with a similar incidence in European countries with a predominantly Caucasian population [2]. An earlier or younger age of diagnosis of UM has been associated with a more favourable prognosis [3]. UM can affect any part of the uveal tract, but choroidal melanoma (CM) is more predominant (86.3%), while iris and ciliary body (CB) melanomas are far less frequent [4]. The age of most patients with UM ranges from 50 to 80 years, with a peak in the seventies and a mean age of 58 years [4,5]. Survival of UM patients has been consistently poor [6,7] possibly due to the silent hematogenous spread even before clinical evidence and diagnosis of the ocular UM, ultimately resulting in systemic micro-metastases [8,9]. Histopathological bad prognostic indicators have been identified and extensively studied. These include large basal tumor diameter, epithelioid cell type, high mitotic activity, CB involvement, and the presence of closed extravascular matrix loop [10]. Although three decades ago the major debate in the management of UM dealt with validating the efficacy of eye-sparing treatment for medium size tumors, the current trends look to expand eye-conserving therapies to larger tumors and to promote early therapy of smaller tumors aimed at preventing tumor growth and subsequent mortality [11,12]. With the recent insights into the genetics and immunology of this rare cancer, the role of molecular testing will grow as tailored therapies, and early treatment of the metastatic disease become more feasible [13].

Many articles have been published in relation to the importance of genetic testing in uveal melanoma [14,15]. Prescher in 1996 reported the earliest historical observation predicting worse prognosis in UMs that show chromosome 3 monosomy [16]. In that retrospective report, 54 enucleated globes with uveal melanoma were evaluated to investigate the correlation of the copy number of chromosomes 3 to the patients’ outcome [16]. Several publications on genetic testing of melanoma from enucleated eyes have confirmed their observations [[17], [18], [19], [20], [21]]. Chromosomes 3, and 8 have been more commonly used in relation to prognosis utilizing FISH, high density genome array, and multiplex ligation-dependent probe amplification (MLPA). More specifically, monosomy 3 and polysomy 8 have been highly correlated with metastatic death [10]. Our goal in this study is to evaluate the demographic characteristics of uveal melanoma in our geographic part of the world and to correlate the histopathological features to the limited available cytogenetic analysis for loss of chromosome 3 and gain of chromosome 8.

### 1.1 Patients and methods

The study was approved by the Institutional Review Board and Human Ethics Committee (IRB/HEC) with expedited approval as a retrospective study at King Khaled Eye Specialist Hospital (KKESH) with a form of a collaborative agreement with King Abdulaziz University Hospital (KAUH), Riyadh, Saudi Arabia. A retrospective review (by AA Al Qahtani) of the medical files of 28 patients with choroidal melanoma who underwent enucleation at KKESH and KAUH, between January 2000 and December 2017 was performed. Dual-color fluorescence in situ hybridization (FISH) was performed using centromeric probes for chromosome 3 and 8 in 18/28 of archived patient tissues in King Faisal Specialist Hospital and Research Centre (KFSHRC). A general informed consent was obtained for all cases, including permission for anonymous use of photos and reporting. Inclusion criteria was: all patients with CM regardless of their origin and ethnicity who were treated by enucleation. Exclusion criteria was: cases of CM treated conservatively by modalities other than enucleation. UM primarily involving sites other than the choroid: CB and/or iris.

The parameters studied included gender, age at the time of presentation, and the affected eye. The tumor histological characteristics studied included maximal basal tumor diameter, thickness, shape, tumor pigmentation, an extra-scleral extension of the tumor, and the presence of subretinal fluid as well as the size of the tumor determined by clinical examination. All the 28 specimens in this study were diagnosed as choroidal melanoma by the Pathology Department at KKESH and were all reviewed by 2 the pathologists (HM Alkatan and AMY Maktabi) who are included in this study. Screening for systemic metastasis included the annual examination of liver function tests. Computed tomography or magnetic resonance imaging was used to confirm the metastases that were suspected based on screening examinations.

For cytogenetic analysis, dual-color FISH was performed for chromosomes 3 and 8 on paraffin-embedded tissue blocks from patients who had undergone primary enucleation. Tissue blocks from patients who received any radiation or surgical treatment before their enucleation were excluded from FISH analysis (one patient only received brachytherapy in the involved eye before enucleation). FISH was performed using centromere Enumeration probes for chromosomes 3 and 8 according to the manufacturer's protocol. Briefly, paraffin sections were re-hydrated, air-dried, pretreated and digested with protease before hybridization to fluorescence-labeled probes (orange for chromosome 3 and green for chromosome 8). Follow-up of these patients when available was documented including evidence of metastasis at the time of the last follow-up. Since KKESH and KAUH are tertiary care government facilities, some of them were followed up by their referring hospitals. Hence, long-term follow-up and rate of metastasis and death were not possible for our patients in this study because most of our patients were either lost to follow up or their follow up data was not made available to us. The investigators confirm that this is their original work and no cofounders have been involved. The FISH studies are done as a part of an agreement between the different tertiary centers.

### 1.2 Statistical analysis

Data were collected and stored in a spreadsheet using Microsoft Excel 2010® software. Data were analyzed using SPSS® version 21.0 (*IBM* Inc.^,^ Chicago, Illinois, USA). Descriptive analysis was done, where categorical variables were presented in the form of frequencies and percentages and continuous variables in the form of the mean (±Standard Deviation) and Range (minimum to maximum). Fisher's Exact test was used to compare the proportions between the groups. Any output with a *p* below 0.05 was interpreted as an indicator of statistical significance.

This work has been conducted and prepared for publication in line with the STROCSS guideline (Strengthening the Reporting of Cohort Studies in Surgery) [22]. The Research registry was also performed with registry number:5447.

## 2 Results

The mean and (SD) age of the 28 patients was 56 years (±15.2) ranging from 24 to 84 (median = 56.5) with 13 males (46.4%) and 15 females (53.6%). The majority of the cases were Saudi (92%) with only 2 Non-Saudi patients (7%) as summarized in Table 1. The right eye was mostly affected in 57%. The duration between clinical presentation and enucleation was wide, ranging from 2 days to 61 days with a mean of 6.7 days ± 15.6. None of our patients had lymph node involvement or metastatic disease at presentation. However, 3 patients had abnormal LFT, which was not disease-related. The mean follow-up (FU) time among 27 patients with available FU data was 31.9 months (SD = 34.3) and the FU ranged between 11 days and 122.7 days). There was one detected metastasis upon FU in one patient, which was local metastasis. The 3 patients with abnormal LFT remained stable without progression.

**Table 1:** Demographic data of 28 patients with choroidal melanoma.

Characteristic	N (%)
Age in years, mean ± SD [Range], median	56.1 ± 15.2 [24–84], 56.5
Gender/M:F ratio = 1:1.2	
Male	13 (46.4)
Female	15 (53.6)
Nationality	
Saudi	26 (92.9)
Non-Saudi	2 (7.1)

**Fig. 1:**
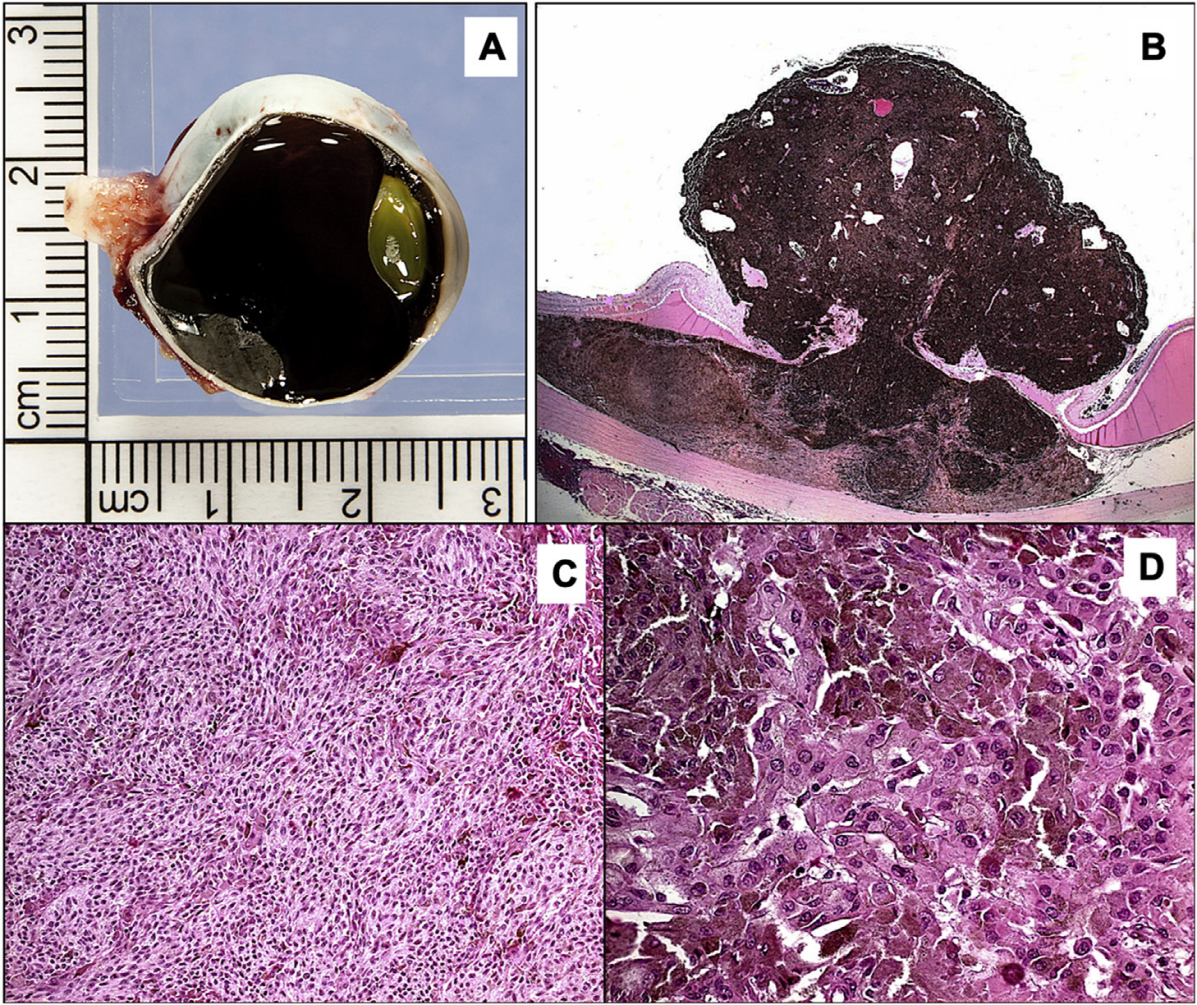
**(A)** The gross photo of a choroidal melanoma. **(B)** Histopathological low power photo of the tumor with the classic mushroom-shaped mass owing to ruptured Bruch's membrane (Original magnification x12.5 Hematoxylin and eosin). **(C)** Histopathological appearance of spindle cell-type melanoma in one area of this mixed cell type tumor (Original magnification x200 Hematoxylin and eosin-bleached). **(D)** Another area with epithelioid cell proliferation (Original magnification x400 Hematoxylin and eosin).

Grossly (Table 2), the tumors attained the classic mushroom shape in less than half of the cases and were amelanotic in 4 eyes only (14.3%). Almost all the patients had subtotal retinal detachment (27/28). The means of the tumor basal diameters were 14.4 mm (SD = 4) and 12.7 mm (SD = 3.8) (ranges: 6.5–25.0 and 6.0–21.0). The mean height was 9.3 mm (SD = 3.8) (range: 2.0–20.0). An example of a mushroom-shaped heavily pigmented UM within the choroid is seen in Fig. 1.

**Table 2:** Gross examination findings in 28 globes with choroidal melanoma.

Characteristic	N (%)
Tumor size:	
a. Tumor base 1 in mm, mean ± SD [Range]	14.4 ± 4.0 [6.5–25.0]
b. Tumor base 2 in mm, mean ± SD [Range]	12.7 ± 3.8 [6.0–21.0]
c. Tumor height in mm, mean ± SD [Range]	9.3 ± 3.8 [2.0–20.0]
Tumor size category	
1	1 (3.6)
2	8 (28.6)
3	12 + 1^a^ (46.4)
4	6 (21.4)
Pigmentation	
Amelanotic	4 (14.3)
Moderate	12 (42.9)
Dark	12 (42.9)
Shape (Mushroom)	
Yes	12 (42.9)
No	16 (57.1)
Retinal detachment	
Yes	27 (96.4)
No	1 (3.6)
Extraocular extension	
Yes	4 (14.3)
No	24 (85.7)

a One patient who had Tumor size category 3 was classified as T4e because of a documented extraocular extension (more than 5 mm).

**Graph 1:**
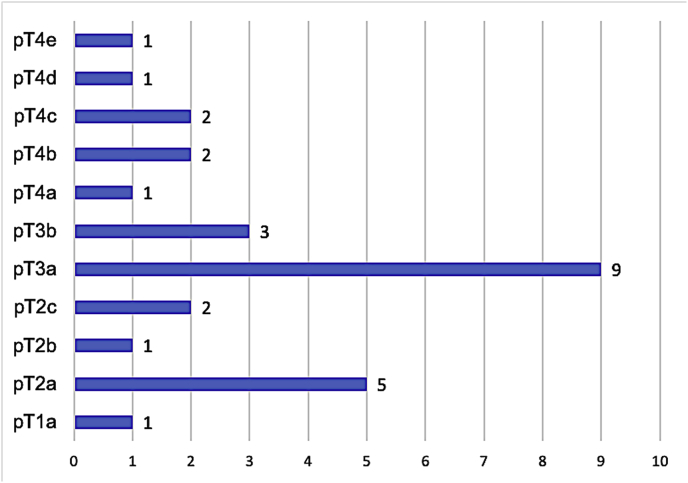
The classification of 28 tumors according to the 8th edition of American Joint Commission for Cancer (AJCC).

**Graph 2:**
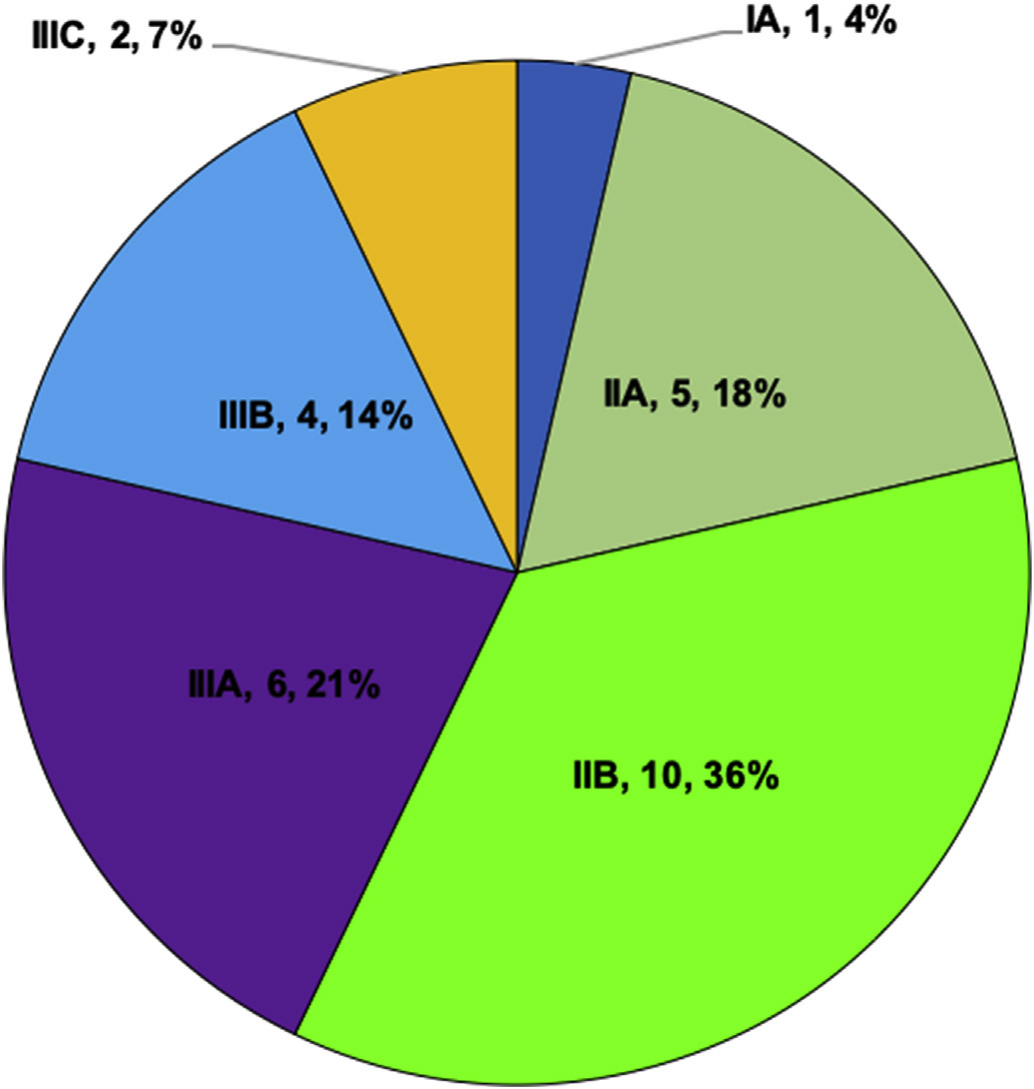
The staging of the tumors in 28 eyes according to 8th edition of the American Joint Committee on Cancer (AJCC).

Histopathologically, the tumor's most common cell type was the spindle type (50%) followed by the epithelioid type (35.7%) and the mixed type (Fig. 1) in 4 eyes only (14.3%). The mitotic index was low with the majority of tumors (53.6) showing one mitotic figure per 40 HPF. Table 3 summarizes the main histopathological features. The tumor extended to involve the CB in 7 eyes (25%). According to the American Joint Committee on Cancer (AJCC) 8th classification, the pathological classification is demonstrated as Graph 1 with the majority being classified as pT3a. Tumor staging is summarized in Graph 2.

**Table 3:** Histopathological findings in 28 globes with choroidal melanoma.

Characteristic	N (%)	
Extension of ciliary body		
Yes		7 (25.0)
No		21 (75.0)
Cell type		
Spindle		14 (50.0)
Epithelioid		10 (35.7)
Mixed		4 (14.3)
Mitotic figures		
1 per 40 HPF		15 (53.6)
2 per 40 HPF		8 (28.6)
3 per 40 HPF		3 (10.7)
4 per 40 HPF		2 (7.1)
Extravascular matrix pattern		
a. Loops		
Present		21 (75.0)
Absent		7 (25.0)
b. Networks		
Present		9 (32.1)
Absent		19 (67.9)
c. Complex pattern		
Present		10 (35.7)
Absent		18 (64.3)
Infiltrating lymphocytes		
None		15 (53.6)
Few		8 (28.6)
Moderate		2 (7.1)
Many		3 (10.7)
Infiltrating macrophages		
None		5 (17.9)
Few		11 (39.3)
Moderate		8 (28.6)
Many		4 (14.3)
Extraocular extension – yes		4 (14.3)
Optic nerve invasion – yes		3 (10.7)

Cytogenetic studies were successfully done for chromosome 3 in 13 eyes and for chromosome 8 in 14 eyes using paraffin-embedded tissue specimens available from the patients who had undergone the primary enucleation. Monosomy 3 was identified in 4 of the 13 cases (30.8%) and the gain in chromosome 8 was detected also in 4 cases. Two cases with monosomy 3 showed a concomitant gain in chromosome 8 as well. We have studied the correlation between the presence of monosomy 3, gain in chromosome 8 or combination of both and the tumor cell type in these eyes. The absence of any genetic abnormality in any of the 2 chromosomes was associated with spindle cell type (*P = 0.005*) and was statistically significant (Table 4). However, the absence of chromosomal abnormality was significantly correlated to higher prognostic staging groups of IIB or worse with *P = 0.004*, which was not expected (Table 5). Finally, we also correlated the presence of chromosomal abnormality to high-risk pathological features: the vascular pattern, lymphocytic and macrophage infiltrate. Table 6 revealed a statistically significant correlation between the presence of vascular loops and chromosomal 3 or 8 abnormalities (*P = 0.027*) while in contrary, absence of any chromosomal abnormality was correlated with the presence of vascular networks (*P = 0.004*).

**Table 4:** Correlation between either chromosome 3 loss OR chromosome 8 gain OR combined abnormality in both chromosomes with the histopathologic cell type in 14 patients where FISH was performed.

Cell type	No loss/gain	Abnormality
Spindle	6	2
Epithelioid	2	3
Mixed	0	1
Total	8^a^	6

Abnormality spindle (2/6) vs epithelioid + mixed (4/6) = 0.268.

a P value comparing spindle (6/8) with epithelioid + mixed (2/8); no loss/gain = 0.005*.

**Table 5:** Correlation of having either Chromosome 3 loss OR Chromosome 8 gain or combined abnormality with Tumor-Anatomic/Prognostic Staging groups in 14 patients where FISH was performed.

Anatomic/prognostic Stage	No loss/gain	Abnormality
IA	0	0
IIA	1	2
IIB	4	1
IIIA	2	1
IIIB	1	1
IIIC	0	1
**Total**	8	6

No loss/gain IA and IIA = 1/8 vs IIB and worse = 7/8; p value = 0.004*.

Abnormality IA and IIA = 2/6 vs IIB and worse = 4/6; p value = 0.268.

**Table 6:** Correlation between the chromosomal abnormality, vascular pattern, lymphocytic infiltrate, and macrophage infiltrate.

High risk feature	No loss Ch 3	Abnormality Ch 3	No gain Ch 8	Abnormality Ch 8	No loss Ch 3 OR Gain Ch 8	P value	Abnormality Ch 3/Ch 8	P value
Vascular pattern								
Loops								
Present	6	4	7	3	5	0.333	5	0.027^a^
Absent	3	0	3	1	3		1	
Networks								
Present	1	2	1	2	1	0.004^a^	2	0.268
Absent	8	2	9	2	7		4	
Complex pattern								
Present	3	2	4	2	3	0.333	3	0.998
Absent	6	2	6	2	5		3	
**Lymphocytes infiltrate**								
None	5	3	6	2	4	0.998	4	0.268
Present	4	1	4	2	4		2	
**Macrophages infiltrate**								
None	3	1	3	1	2	0.053	2	0.268
Present	6	3	7	3	6		4	

Ch: Chromosome.

a Statistically significant at 5% level of significance.

## 3 Discussion

Uveal melanoma (UM) represents 5% of all melanomas with an age-adjusted risk of 5 per 1 million in the United States [23]. In Caucasians, the incidence of UM ranges from under 2 to over 8 per million annually but these tumors are less common in races with brown eyes [2,24]. In a large analysis of 8033 cases of uveal melanoma, the racial distribution was primarily in Caucasians (98%) [25]. The mean age at the time of UM diagnosis in that study was 58 years (with a range of 3–99 years) and a majority (53%) were in mid-adults 21–60 years, 45% in older adults and only 1% of UM was diagnosed in patients below 20 years of age [25]. In our study, the mean age was 61 but we were unable to comment on the prevalence and incidence of UM considering our relatively small number of cases and that as per our ethnicity, our study had significant population bias. The UM does not seem to have noticeable gender predilection; however, we have observed slight female predominance in our study (54%) [25]. The choroid is the most common location in 90% of cases, with the rest being in the ciliary body or the iris [25,26]. Risk factors for developing UM are believed to be the presence of a pre-existing choroidal nevus and oculo-dermal melanocytosis known as nevus of Ota [27]. Regarding tumor size, Meta-analysis of Diener-West et al. attempted to provide systematic results of eight studies on mortality rates following the enucleation of UM [28]. For small (<3 mm-thick and <10 mm in basal diameter), medium (3–8 mm-thick and <15 mm in basal diameter) and large (>8 mm-thick and >15 mm in basal diameter) tumors, 5-year overall mortality was 16%, 32%, and 53%, respectively [28]. More recently, Brovkina concluded a higher risk of hematogenous spread with large size choroidal melanomas with metastatic disease developing in every fifth patient with UM larger than 15 mm [29]. Shields et al. adopted tumor thickness as the criterion of tumor size; they decided that the acquisition of this dimension by ultrasonography ensures higher precision than the measurement of basal tumor diameter [5]. In our study, almost half of our cases had a tumor size category 3 (46.4%) and the larger tumor size categories of 3 and 4 collectively constituted approximately 68% of the cases. Also, we had only 1 patient who had local recurrence with a basal diameter of 20 mm and a height of 18 mm, which was considered a large tumor. Even though our study did not correlate the tumor size directly to prognosis, since we did not have long term follow up, we observed that tumor size category 1 and category 2 without CB involvement shown as AJCC stage grouping IA and IIA were significantly associated with absent chromosomal abnormality being detected (*P = 0.004*) and thus an overall better prognosis. This combined importance of tumor size reflected upon the AJCC staging, together with the genetic status, has been also clarified by Bagger where the frequency of tumors with normal genetic testing decreased with increasing 7th AJCC staging [29]. They also concluded that combined stage III and abnormal Chromosome 3 and 8 copy numbers were considered as significant predictors for poor prognosis in their multivariate Cox regression analysis [30].

The histopathologic cell type of UM morphologically is important. The Spindle type of UM shows elongated cells with large nuclei and scant cytoplasm (low nuclear to cytoplasmic ratio). They are uniformly and densely arranged and they may form palisades. There are very few cells with prominent nucleoli, and any mitotic figures are hardly observed. Epithelioid cell type is characterized by larger cells with abundant acidophilic cytoplasm, large round or oval nuclei, a high nuclear to-cytoplasmic ratio and a high number of mitotic figures. Tumors with epithelioid cell type have been related to a higher probability of developing UM metastasis and a higher rate of mortality [31]. The epithelioid cell type comprises approximately 3–5% of all UM and it is associated with the least favourable prognosis. The 15-year mortality rate among patients diagnosed with epithelioid cell type UM is 75% [32]. Spindle cell type accounts for approximately 40% of all UM. The 15-year mortality rate is 20% [32]. The mixed type is the most frequent one and it represents up to 50% of all UM. The 15-year mortality rate is approximately 60% but considerable differences are observed depending on the percentage of epithelioid and spindle cells [32]. In our study, the commonest cell type was the spindle in 50%. Also, the absence of cytogenetic abnormality in chromosome 3 and/or chromosome 8 was significantly associated with spindle cell type tumors as shown in Table 4 which supports an expected better prognosis in these patients.

Folberg described in depth the morphological patterns of extravascular matrix in UM related to the presence of fibrous septal networks separating the blood vessels that are present between the collection of tumor cells. These vascular patterns were best assessed using Periodic acid-Schiff staining and were a subject of controversy [33,34,35]. Others described two patterns, namely loops and networks. The loop pattern was identified in 60% of cases and the network pattern by 35% in one of the studies and the prognosis of tumors with a network pattern and of those with loops did not differ significantly [36]. On the other hand, in another study, Lee identified the presence of closed extravascular matrix loop as a predictor factor for melanoma-related mortality [10]. In our study the presence of closed vascular loops -rather than networks-was significantly associated with abnormal cytogenetic studies of chromosomes 3 (3 loss) and 8 (q gain), and thus had expectations of a worse prognosis (*P = 0.027*). In contrast to that, having vascular networks in the tumor seems to be a good prognostic indicator with a statistically significant absence of chromosomal abnormalities in that group (*P = 0.004*). We did not manage to find any significant associations between the cytogenetic results and inflammatory cell infiltration. Worse prognosis in UM has been observed in association with inflammatory infiltration by an increasing number of lymphocytes, macrophages, as well as human leukocyte antigen (HLA) I and HLA II expression [37]. However, no statistically significant difference in mortality was demonstrated concerning this [38]. A considerable percentage of macrophages in the lymphocytic infiltration has been also correlated with other factors such as: female sex, the tumor largest basal diameter, an epithelioid cell type, strong pigmentation, microvascular density, and metastasis-related mortality [39].

Common sites of UM metastases include liver (90%), lungs (24%) and bones (16%), with multiple occult metastases seen on autopsy [[40], [41], [42]]. The Collaborative Ocular Melanoma Study (COMS) protocol advocates a 5-years monitoring of chest radiographs and liver function tests every 6 months [43]. Abnormal liver function tests were found to be highly specific (92%) but had a sensitivity of less than 15% in the diagnosis of metastatic uveal melanoma [41]. In our study, we had 3 patients with tumor-unrelated abnormal LFT that persisted after enucleation and one patient with local metastasis following enucleation.

**Fig. 2:**
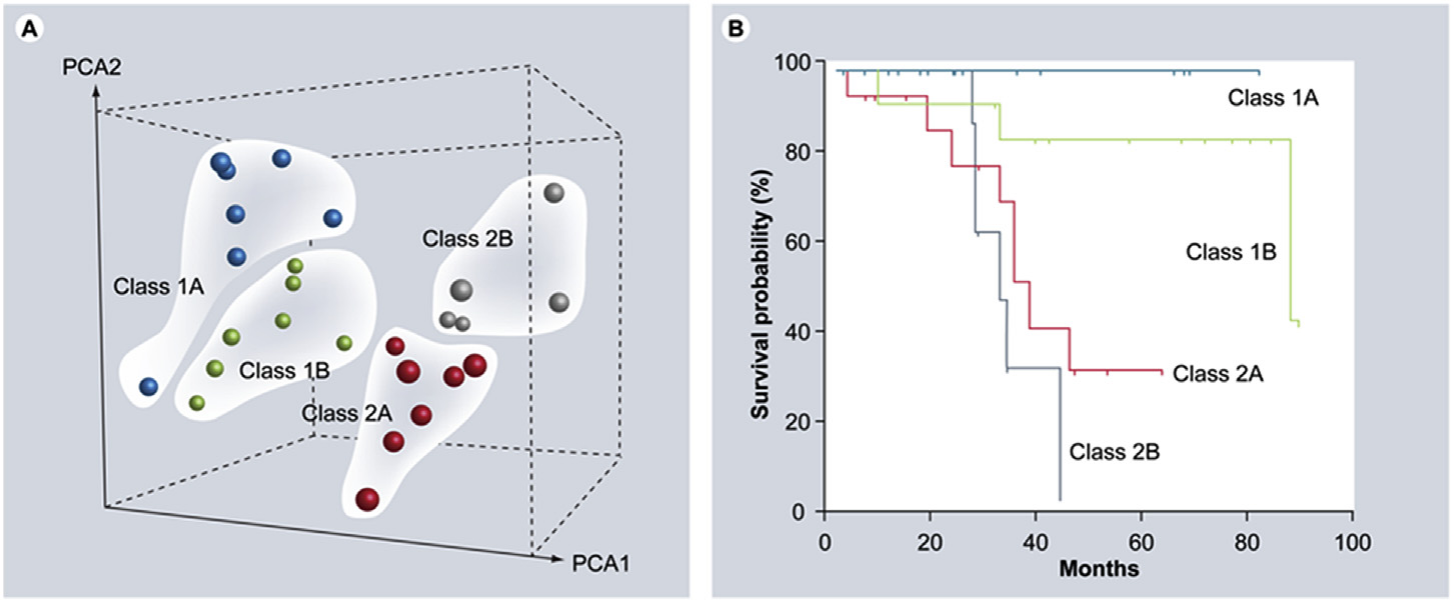
**Molecular classification of uveal melanomas based on transcriptomic and chromosomal features. (**Reproduced with permission from Future Medicine, Contract # FMQ-42553/1) (A) Unsupervised principal component analysis, showing natural clustering of uveal melanomas into four groups according to gene-expression profile and status of chromosomes 3, 6p and 8p. Class 1A – minimal aneuploidy (blue spheres); class 1B – 6p gain (green spheres); class 2A – monosomy 3 (red spheres) and class 2B – monosomy 3 and 8p loss (gray spheres). **(B)** Kaplan–Meier survival analysis showing that molecular classification accurately predicts metastatic death. PCA: Principle component analysis. (For interpretation of the references to color in this figure legend, the reader is referred to the Web version of this article.)

Cytogenetic and molecular genetic studies are generally of paramount importance in the prediction of UM prognosis. The original work using DNA evaluation has generally demonstrated the association of the loss in chromosome 3? and the gain in chromosome 8 with decreased survival [25,44]. Furthermore, it has been demonstrated using MLPA that the 10-year predictive melanoma-related mortality was reported to be 55% for cases with monosomy 3 and 71% for cases with combined monosomy 3 and chromosome 8 gain [45]. Even though MLPA is the preferred method by some for genetic testing, they have also advocated the use of microsatellite analysis of chromosome 3 if DNA yield is less than 100 ng from intraocular biopsies. They have shown similar prognostic information and proved its usefulness [46]. In our part of the world, many patients are reluctant to undergo a biopsy of the UM for prediction of prognosis, therefore genetic testing is performed using FISH on tumors following enucleation and we lack genetic information on cases treated by other modalities. Others have employed RNA genetic evaluation and confirmed the presence of 2 major classes of melanoma: a low-grade class 1 with 95% 8-years survival and a high-grade class 2 with 31% survival [20]. A more complex molecular-based prognostic classification with further work to subdivide the previously described classes into four subclasses (1A, 1B, 2A and 2B) with significant prognostic value, based on gene-expression profiling has followed and was strongly advocated as demonstrated in Fig. 2 [47].

The AJCC ophthalmic oncology task force has validated the AJCC 7th classification of UM with the demonstration of 5- and 10-year Kaplan-Meier metastasis-free estimates of 97% and 94% for stage I compared to 50% (for both 5- and 10-year) in cases with stage IIIB [48].

In our study, we have used the 8th edition of the AJCC classification and have correlated the staging to our genetic results however, the relatively small number of available genetic testing had negatively affected the successful correlations mentioned earlier between the cytogenetic results and the AJCC tumor staging. Other limitations of our current retrospective study are the small sample size, the limited genetic testing to FISH analysis only in addition to the lack of genetic profiling information from tumors managed by other treatment modalities (since patients tend to deny diagnostic incisional biopsies), and finally the limited follow-up information.

## 4 Conclusions

We observed a relatively low incidence of UM in our study compared to the Caucasian populations despite the mixture of ethnicity groups in our country. However, we also observed the tendency for late presentation resulting in significant visual morbidity, larger tumor growth, and possibly a higher rate of enucleations. This study provided us with interesting conclusions. The spindle cell type was also significantly associated with the absence of cytogenetic abnormality in chromosome 3 and/or chromosome 8, while closed vascular loops were significantly associated with abnormal results of chromosomes 3 and 8. The development of our new national tumor registry should help identify new cases of UMs to improve our database. We also need to facilitate more genetic testing for UM in Saudi Arabia to be able to study the outcome and disease-related survival in these cases.

## Declaration statement

This study was prepared in accordance with the ethical standards of the human ethics committee at KKESH and expedited approval as a retrospective study (RP 1704-R) from the HEC/IRB of the Research department in accordance with the Helsinki Declaration and with a collaborative agreement with KAUH. A general informed consent was taken from all cases which includes permission for anonymous use of photos and reporting. International Research registry #5447.

## Funding

This work did not receive funding from any of the institutions.

## Declaration of competing interest

The authors have no conflict of interest or financial disclosures in relation to this work.
